# CBCT-Based Online Adaptive, Ultra-Hypofractionated Radiotherapy for Prostate Cancer: First Clinical Experiences

**DOI:** 10.3390/medicina61101839

**Published:** 2025-10-14

**Authors:** Georg Wurschi, Alexander Voigt, Noreen Murr, Cora Riede, Michael Schwedas, Maximilian Römer, Sonia Drozdz, Klaus Pietschmann

**Affiliations:** 1 Department of Radiation Oncology, Jena University Hospital, 07747 Jena, Germany; 2 Comprehensive Cancer Center Central Germany, Partner Site Jena, 07747 Jena, Germany; 3 Interdisciplinary Center for Clinical Research, Jena University Hospital, 07747 Jena, Germany

**Keywords:** online adaptive radiotherapy, CBCT, ultra-hypofractionated radiotherapy, prostate cancer

## Abstract

*Background and Objectives*: Ultra-hypofractionated radiotherapy (uhRT) is increasingly used for low- and intermediate-risk localized prostate cancer, necessitating exceptional precision compared to conventional fractionation. CBCT-based online-adaptive uhRT may help mitigate pelvic organ motion but has not yet been established in clinical routine. We report initial clinical experiences focusing on the feasibility and technical aspects of treatment delivery. *Materials and Methods*: Seven patients (35 fractions) with low- or intermediate-risk prostate cancer were treated with online-adaptive uhRT on the Varian Ethos^®^ system within routine clinical care. The target included the prostate and proximal seminal vesicles (CTV1, 5 × 7.25 Gy), with an integrated boost to the prostate (CTV2, 5 × 8.00 Gy). For each fraction, dose–volume histogram (DVH) parameters for targets and organs at risk (OARs) were recorded retrospectively for both scheduled and adaptive plans, along with the plan selection decision. Plan quality was evaluated per clinical DVH constraints and target coverage. The treatment time was recorded. *Results*: Online-adaptive uhRT was successfully delivered every day in 5 patients and on alternate days in 2 patients. Mean treatment time was 30:17 (±05:49 SD) minutes per fraction. The median recorded change in target and OAR volumes was <10%. Adaptive plans resulted in a statistically significantly improved target coverage for CTV1 (V100%, *p* = 0.01), PTV1 (D98%, *p* < 0.001), PTV2 boost (D98%, *p* < 0.001) in Wilcoxon signed-rank tests. OAR dose reduction was limited, with a small improvement in bladder V40Gy (*p* = 0.02). Adaptive plans were applied in 32/35 fractions (91.4%). To encompass intra-fractional motion in 95% of fractions, positional adjustments up to 0.77 cm (longitudinal), 0.37 cm (lateral), and 0.59 cm (sagittal) were required. *Conclusions*: Online-adaptive uhRT appears feasible, leading to optimized target volume coverage. Considerable treatment times must be taken into account. A second CBCT is recommended to compensate for intra-fractional motion. Further research regarding patient-related endpoints and cost-effectiveness is highly needed.

## 1. Introduction

Ultra-hypofractionated radiotherapy (uhRT), delivered in just 5 to 7 fractions, has gained recognition as a clinically equivalent alternative to conventional regimens involving 20 to 40 sessions [1,2,3]. Beyond preserving therapeutic efficacy, this approach significantly shortens treatment duration and supports a more efficient and sustainable use of healthcare resources [4,5,6]. The application of higher doses within a substantially lower number of fractions demands precise treatment delivery. Inter- and intra-fractional anatomical variability particularly involves adjacent organs, such as the bladder and rectum, increasing the risk of toxicity. Traditional strategies to reduce this variability, such as bowel preparation and standardized bladder filling protocols, may be burdensome for patients and offer only modest improvements in reproducibility [7]. The implementation of online-adaptive radiotherapy (oART) represents a paradigm shift in prostate cancer treatment. By facilitating daily, image-guided plan adaptation based on actual anatomical conditions, oART enhances dosimetric precision, improves target conformity, and may reduce exposure to surrounding organs at risk (OAR) [8,9]. Moreover, it obviates the need for extensive patient preparation protocols, thereby streamlining workflow and improving the overall treatment experience. oART has been provided to patients undergoing normofractionated or moderately hypofractionated RT by different working groups on magnetic resonance imaging (MRI)-based [10,11,12,13] or computed tomography (CT)-based systems [14,15], demonstrating improved target volume coverage and OAR sparing. Given the relatively long image acquisition times on MR-based systems, CBCT-based treatments appeared favorable regarding time-dependent confounders, such as anatomic shifts caused by bladder and rectal filling [16]. The feasibility of so-called “plan-of-the-day” approaches with reoptimization for the current anatomy has successfully been demonstrated with conventional linear accelerators (LINACs) [17,18] and cone beam CT (CBCT)-based oART with dedicated systems such as the Varian Ethos^®^ LINAC [19,20]. The recent introduction of high-resolution ring-gantry CBCT imaging systems facilitates the daily adaptation process [21]. However, there is still limited published data on the implementation of a standardized oART workflow in routine clinical practice. This case series aims to address this gap by presenting practical insights and clinical experiences with the CBCT-based Varian Ethos^®^ platform.

This report outlines our initial clinical experience with online-adaptive uhRT for prostate cancer on a Varian Ethos^®^ system. The primary focus was on technical feasibility and clinical workflow efficiency, particularly in comparing adapted plans to non-adapted (‘scheduled’) plans. Our hypothesis was that selecting adapted plans within the clinical workflow would improve target coverage and OAR sparing. Furthermore, we assessed the time required for online adaptation and the anatomical variations occurring during this interval.

## 2. Materials and Methods

### 2.1. Study Design

We conducted a monocentric retrospective observational study. All included patients had histologically proven low or intermediate risk localized prostate adenocarcinoma and were >18 years old. A curative-intent radiotherapy recommendation was provided in each case by a multidisciplinary tumor board prior to treatment. Within a pretreatment visit, suitable fractionation schemes and radiotherapy techniques were offered and patients actively opted for online-adaptive uhRT. Treatments were delivered between February and April 2025 at Jena University Hospital. This study was reported in accordance with the STROBE recommendations [22] for observational research.

### 2.2. Patient Preparation and CT Simulation

All patients were instructed and trained to comply with an institutional hydration protocol, aiming for sufficient bladder volume (>150 mL), and with dietary recommendations to avoid gas-producing foods. Patients were immobilized in the supine position using institutional setup devices, and planning CT scans (SOMATOM Go Open Pro, Siemens Healthineers, Erlangen, Germany) were acquired with a 2 mm slice thickness. Image acquisition was restricted to the anatomical region relevant for dose calculation to streamline subsequent on-couch adaptation. No intravenous contrast or fiducial markers were used. Previously acquired multiparametric magnetic resonance imaging (mpMRI) sequences were co-registered with the planning-CT to facilitate accurate anatomical delineation.

### 2.3. Treatment Planning

All imaging datasets were transferred to the Ethos^®^ v2.0 treatment planning system (Varian Medical Systems, Palo Alto, CA, USA) for target and OAR delineation. Delineation was performed by a board-certified radiation oncologist with at least ≥7 years of clinical experience and independently reviewed by a second specialist. The clinical target volume (CTV1) included the prostate and proximal seminal vesicles (1 cm). A simultaneous integrated boost (SIB) volume (CTV2) was defined, including the prostate only. OARs comprised the bladder, rectum, bowel (including the sigmoid colon), penile bulb and femoral heads. All structures were delineated prior to adaptive treatment sessions based on the planning CT. The planning target volume (PTV) was derived from the CTV1 using a 5 mm isotropic margin, except posteriorly, where a 3 mm margin was applied to reduce rectal dose. Following the PACE-B protocol [23], no additional PTV margin was applied for PTV2 (=CTV2) in order to prioritize OAR sparing. Both PTVs were re-generated at each fraction, allowing for daily adaptive re-contouring of the CTV while maintaining consistent margins. A total dose of 40 Gy in five fractions (8 Gy per fraction) was prescribed to CTV2, and 36.25 Gy in five fractions (7.25 Gy per fraction) to the PTV1 [23,24]. Radiotherapy was delivered using 12-field intensity-modulated radiotherapy (IMRT). Volumetric modulated arc therapy (VMAT) was not employed due to longer optimization and calculation times, and thus was not integrated into clinical routine at the time of treatment. Dose–volume constraints for target volumes and OARs were based on the PACE-B trial [24] and used to generate a standardized treatment planning template (Appendix A.1).

### 2.4. Online Adaptive Radiotherapy Workflow

**Figure 1:**
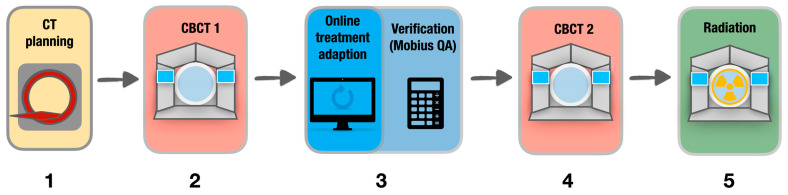
The applied workflow during an online-adaptive treatment session: A planning computed tomography (CT) scan is acquired prior to treatment and serves as the reference for initial planning (**1**). A daily cone beam CT (CBCT 1) is obtained at the beginning of each treatment session (**2**). Daily anatomical variations are delineated on this image and used for online-adaptive treatment planning. An independent verification software (Mobius 3D v4, Varian Medical Systems, Palo Alto, CA, USA) is employed to validate the adaptive plans prior to treatment (**3**). A second CBCT 2 (**4**) is used to account for intra-fraction motion during the planning period, and the online-adaptive treatment fraction is delivered immediately afterward (**5**).

Treatments were delivered either daily (q1d) or on alternate days (q2d), based on individual patient preference and physician discretion, following the workflow depicted in Figure 1.

**Figure 2:**
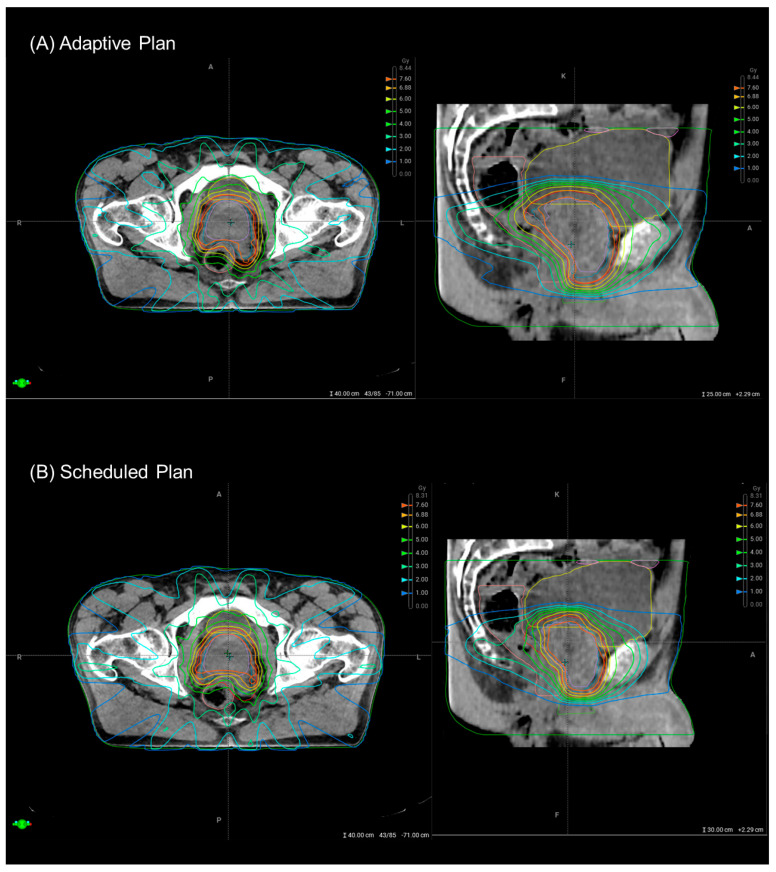
Comparison between an exemplary adaptive plan (**A**), based on the adaptation of structures in the current cone beam computed tomography (CBCT), and the corresponding scheduled plan (**B**), in which the initial dose distribution is superimposed onto the anatomy of the current CBCT. The adapted plan enables improved coverage of the planning target volume (PTV1, red), which includes the prostate and proximal seminal vesicles, particularly in posterior regions adjacent to the rectum (pink). At the same time, doses to the bladder (yellow) are reduced. The displayed isodose lines correspond to 95% of the prescribed dose for PTV1 (7.25 Gy) and PTV2 (boost to 8.00 Gy, light red structure comprising the prostate only).

Prior to each fraction, patients followed the standardized hydration and dietary protocol as previously described. Patients were positioned supine using institution-specific immobilization devices to ensure reproducibility. At each treatment session, a pre-treatment cone beam CT (CBCT1) was acquired. The Ethos^®^ adaptive RT platform employed artificial intelligence (AI)–driven auto-segmentation to delineate key anatomical structures (‘influencers’) based on the OAR defined above. All influencer contours were reviewed by an experienced radiation oncologist and manually adjusted if necessary. The PTV was derived from the CTV automatically re-generated using the predefined anisotropic margins (+3 mm posterior, +5 mm in other directions) at each fraction without manual adjustment. Using predefined clinical objectives, following the template provided in Appendix A.1, and a standardized 12-field IMRT beam configuration, the Ethos^®^ system generated two plans per fraction: a so-called “scheduled plan”, in which the initial dose distribution is superimposed onto the anatomy of the current CBCT, and a fully re-optimized adaptive plan based on the current anatomy (Figure 2).

An independent verification of the dose distribution was performed using simulation in the Mobius 3D quality assurance (QA) software v4 (Varian Medical Systems, Palo Alto, CA, USA) via gamma analysis (3%/3 mm criteria). Both plans underwent clinical review. In cases where the adaptive plan demonstrated superior target coverage or improved sparing of organs at risk (OARs), it was selected for treatment. A second CBCT (CBCT2) was performed immediately prior to beam delivery to verify intra-fractional anatomical consistency. Couch corrections were applied as needed; the respective couch shifts were recorded to assess the magnitude of intra-fraction motion. For evaluation of feasibility and efficacy, treatment times were recorded based on the log files within the Ethos^®^ treatment planning system.

Toxicity was assessed eight weeks after completion of treatment according to CTCAE version 5.0.

### 2.5. Statistical Analysis and Visualization

Patient characteristics and a detailed overview of adaptive and scheduled plans are reported descriptively. Adaptive plans and their corresponding scheduled plans were compared in an exploratory analysis with respect to target volume coverage and organ-at-risk (OAR) doses, using paired-sample Wilcoxon signed-rank tests. For all tests, the alternative hypothesis specified a directional effect: Measure 1 was hypothesized to be either greater than or less than Measure 2, depending on clinical intent. Regarding target volume coverage, for example, it was hypothesized that CTV1 V100% (adaptive) is greater than CTV1 V100% (scheduled). In contrast, for OAR doses, the hypothesis was that bladder D40% (adaptive) is lower than bladder D40% (scheduled). *p*-values < 0.05 were deemed statistically significant, as no correction for multiple testing was applied in this exploratory analysis. All analyses were carried out with Excel v16 (Microsoft Corporation, Redmond, WA, USA) as well as JASP v0.19.3 (JASP Team, 2025, University of Amsterdam, Amsterdam, The Netherlands [25]); additional illustrations were created with Keynote v14.3 (Apple Inc., Cupertino, CA, USA).

## 3. Results

A total of 7 men with a median age of 67 (Q1–Q3: 65.5–71.5) years were included in this analysis. All patients were diagnosed with localized (cN0 cM0) prostate cancer, which was classified as low risk (n = 2), intermediate favorable (n = 1), and intermediate unfavorable (n = 4) according to the NCCN criteria [3], respectively, and were in good general health condition (Eastern Cooperative Oncology Group, ECOG, performance status 0). The mean prostate volume was 60.7 ± 13.7 (SD) cm^3^. Detailed characteristics are provided in Table 1 and Appendix A.2. Online adaptive uhRT was successfully completed as planned, resulting in the analysis of 35 individual treatment sessions. Daily treatment was administered to 5 patients, while 2 patients received treatment on alternate days. DVH parameters of the reference plan, which was based on the planning CT, met the predefined clinical goals and are provided in Appendix A.3.

During treatment, target volumes as well as OAR volumes measured on CBCT1 demonstrated considerable intra-individual variability, as provided in detail in Appendix A.4 and Appendix A.5. The median intra-individual relative volume changes across all recorded fractions was <10% and for the third quartile, i.e., 75% percentile, <20%, respectively. Extreme variation was observed in bladder (minimum −84.6%, maximum +75.7%) and rectum (minimum −53.8%, maximum +84.8%) volumes. The smallest variation was observed within the CTV2 volume, i.e., the prostate GTV (median: +0.55%; Q1–Q3: −5.5% to +6.9%).

**Figure 3:**
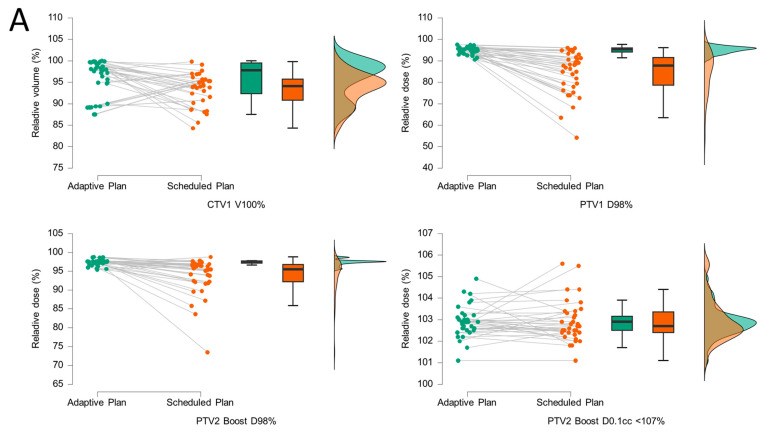

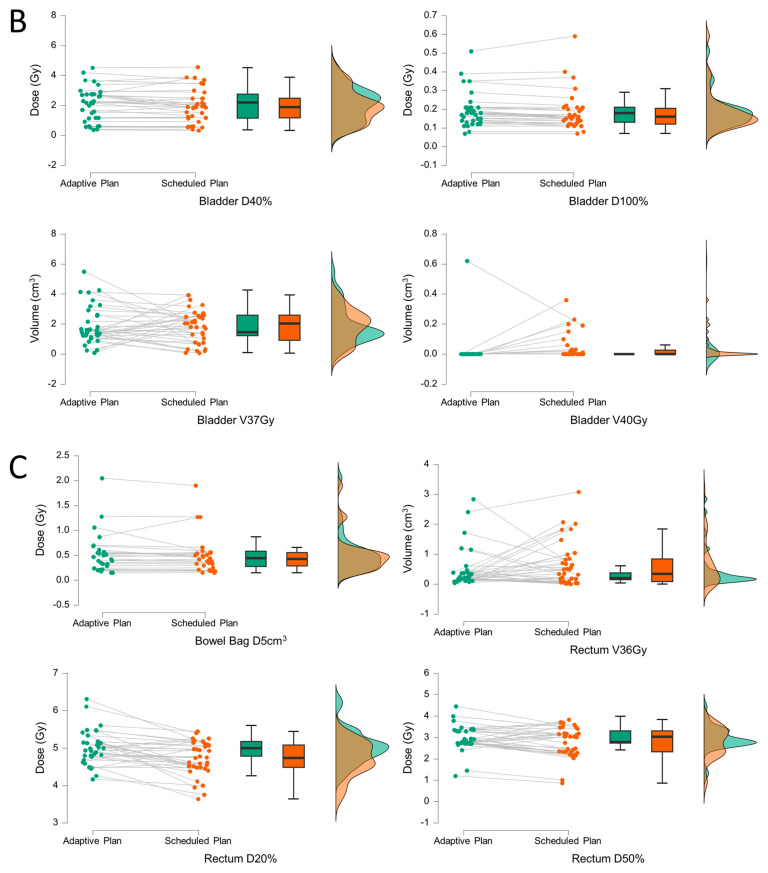

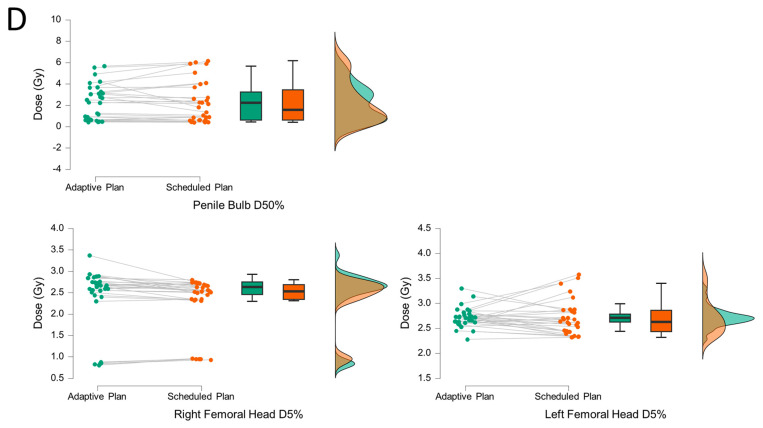
Raincloud and box plot comparison of adaptive (green) and scheduled (orange) treatment plans with respect to key dose–volume histogram (DVH) parameters. Target volume parameters for CTV1, PTV1, and PTV2 (boost) are depicted in panel (**A**), while organ-at-risk (OAR) dose metrics are shown in panels (**B**–**D**). DVH metrics are reported either as dose values corresponding to specific volumes (Dxx%/Dxx cm^3^) or as volume percentages or absolute volumes receiving defined dose thresholds (Vyy Gy/Vyy%) per fraction.

The adaptive and scheduled plans were compared for each fraction (n = 35) using graphical representation (Figure 3) and Wilcoxon signed-rank tests (Table 2). The corresponding dose–volume histogram (DVH) statistics of the targets and OAR can be found in Appendix A.6. A statistically significant improvement was observed in the coverage of CTV1 (V100%, *p* = 0.01), PTV1 (D98%, *p* < 0.001), and PTV2 boost (D98%, *p* < 0.001). Notably, dose “hot spots” within the boost volumes were not more frequent in the adaptive plans (PTV2 D0.1cc < 107%, *p* = 0.38, Table 2A). Overall, CTV1 V100% was better in 23/35 (65.7%) fractions, PTV1 D98% in 34/35 (97.1%) fractions and PTV2 (Boost) D98% in 30/35 (85.7%) fractions, respectively. With the exception of high-dose areas within the bladder (V40Gy, *p* = 0.02), no statistically significant reductions in OAR doses were observed in this cohort (Table 2B). The adaptive plan was chosen in 32/35 fractions (91.4%) due to improved target coverage (30/35, 85.7%) and/or reduced OAR dose (12/35, 34.3%). The reasons for selection of the scheduled plan were reduced OAR doses (2/35, 5.7%) or a lack of DVH improvement with the adaptive plan (1/35, 2.9%).

We further evaluated intra-fractional motion based on the couch shifts, which were applied prior to treatment delivery based on target displacements detected in CBCT2 (Table 3). Although mean treatment shifts were below 5 mm in all directions, a positional adjustment range of up to 0.77 cm in the longitudinal (−0.58 cm caudal, +0.19 cm cranial), 0.37 cm in the lateral (−0.21 cm right, +0.16 cm left), and 0.59 cm in the sagittal (−0.48 cm posterior, +0.11 cm anterior) directions was necessary to encompass 95% of all treatment fractions.

We documented a mean treatment time of 30:17 ± 5:49 (SD) min per fraction (Table 4). The contouring/adaptation process accounted for approximately one-third of the mean total duration (12:11 ± 5:14 (SD) min). The QA tests added an average of 5:27 min ± 1:23 min (SD) to the total treatment time. There was no statistically significant correlation between the length of certain treatment periods and couch shifts (*p* ≥ 0.05, Appendix A.7).

Acute toxicity grade 1 or 2 occurred in 5 out of 7 patients (71.4%), as shown in Table 5. No grade ≥ 3 events were observed. The most frequent toxicities were cystitis and proctitis in each 4 patients (57.1%).

## 4. Discussion

To the best of our knowledge, this is one of the first reports on online CBCT-based, online adaptive uhRT in routine clinical practice. So far, we have identified only one single case report [20] and a small retrospective case series [19], both involving heterogeneous treatment regimens. In the latter, patients also received online adaptive uhRT according to the PACE-B protocol; however, the use of additional fields to pelvic lymph nodes and the inclusion of various cancer stages limit comparability. While our report largely confirms the initial findings of Waters et al. [19], it provides novel insights regarding treatment time, thereby contributing to the assessment of feasibility in a clinical setting.

We demonstrated a benefit in target volume coverage when using adaptive plans compared to scheduled plans. However, except for bladder D40%, OAR doses did not differ significantly in this relatively small cohort. Waters et al. also reported improved target volume coverage, but they found a reduced mean rectal D0.03cc in adaptive plans [19]. Larger studies are needed to confirm these effects in more robust patient samples. Given the consistently high CTV1 coverage (V100%) across nearly all fractions, a systematic reduction in CTV-to-PTV margins should be further investigated. Within our cohort, the adaptive plan was preferred over the scheduled plan in nearly all cases due to superior target coverage. These findings align with reports from oART for prostate cancer with conventional or moderately hypofractionated schedules [9,14,19,26]. Byrne et al. reported treatment times per fraction of 34 min on average in normofractionated and moderately hypofractionated oART [26], which are comparable to those in our uhRT cohort. Given the substantially reduced number of fractions in ultra-hypofractionated schedules and the comparable time per fraction, online-adaptive uhRT is likely to be more time-efficient overall. However, the current evidence still remains too limited to draw definitive comparisons.

The observed range of couch shifts exceeds commonly applied PTV margins in prostate RT, emphasizing the necessity of a second CBCT to account for intra-fractional motion. Expanding PTV margins to compensate for this motion would counteract the goal of oART, which is to minimize OAR doses. Adaptation time appears to be the most critical factor influencing intra-fractional motion [27]. For bladder cancer, Khouya et al. proposed to add 2 mm to PTV margins per additional 5 min of treatment time [28]. We did not detect any time-dependent effects on couch shifts, although the limited sample size may have reduced the power to identify smaller associations. Up to one quarter of the adaptation time was attributed to the additional QA process required by national radiation safety regulations before treatment delivery. As we did not observe any major gamma test failures during oART in general, we would welcome the opportunity to verify adaptive plans in an offline review setting.

Overall treatment toxicity appeared to be comparable to that observed in the PACE-B trial, which employed the same treatment schedule without online adaptation [24]. However, a direct comparison is not feasible due to the small sample size, as further toxicity comparison was beyond the scope of this analysis. Furthermore, the applied 8-week follow-up interval only captures the delayed flare-up of acute toxicity, and no conclusions regarding late toxicity can be drawn. Longer follow-up is warranted. Finally, the relatively time-consuming oART workflow needs to be evaluated in relation to patient-reported outcomes (PROs) and toxicity data to demonstrate clinical benefit. This aspect is beyond the scope of the present report, focusing on treatment delivery in clinical routine. A prospective multicenter trial (NCT06355050) will be conducted by our working group to compare PROs of online-adaptive uhRT with conventionally fractionated RT. Two phase II trials have already reported good quality of life following MR-guided uhRT [29,30]. However, MR-LINACs are not yet widely available for routine treatment, and longer treatment times (average 45 min [29]) than in our cohort have been reported, possibly increasing the impact of intra-fraction motion [16]. Regarding feasibility in clinical routine, CBCT-guided oART may therefore be preferred over MR-guided oART; however, direct comparisons are currently lacking.

### Limitations

As this report includes only a small retrospective cohort from a single institution for feasibility evaluation, further conclusions are limited. The observed variability in target and OAR volumes might also be attributed to inter-observer variability, as adaptation was not performed by the same physician in all fractions. Inter-observer variability was not assessed due to the small sample size. However, standardized operating procedures (SOPs) for delineation and treatment are used at our institution. Larger prospective studies, particularly those focusing on PROs, are necessary to assess the benefits of online-adaptive uhRT.

## 5. Conclusions

Following the proposed workflow, online adaptive, uhRT is feasible within the clinical routine. Target volume coverage was significantly improved by oART, whereas OAR doses remained largely unchanged in most fractions. A second CBCT immediately prior to treatment delivery is strongly recommended to account for intra-fractional motion.

## Figures and Tables

**Table 1:** Patient characteristics including demographic factors and tumor stage. Radiotherapy (RT) was delivered either daily (q1d) or every other day (q2d).

ID	Age at Start	Gleason Score	Gleason Sum Score	ISUP Grading	Initial PSA (ng/mL)	TNM T Stage	RiskClass (NCCN)	RT Delivery
1	67	3 + 4	7a	2	9.92	2c	intermediate unfavorable	q2d
2	71	3 + 4	7a	2	6.33	2b	intermediate favorable	q1d
3	72	4 + 3	7b	3	4.76	2a	intermediate unfavorable	q1d
4	62	3 + 3	6	1	3.7	2a	low	q2d
5	73	3 + 3	6	1	7.18	2a	low	q1d
6	67	4 + 3	7b	3	7.8	2b	intermediate unfavorable	q1d
7	64	4 + 3	7b	3	15.2	1c	intermediate unfavorable	q1d

**Table 2:** Comparison of adaptive and scheduled plans was performed per fraction using paired samples Wilcoxon signed-rank tests. For all tests, the alternative hypothesis specified a directional effect: Measure 1 was hypothesized to be either greater than or less than Measure 2, depending on clinical intent. Regarding target volume coverage (A), for example, it was hypothesized that CTV1 V100% (adaptive) is greater than CTV1 V100% (scheduled). Conversely, for organ-at-risk doses (B), the hypothesis was that bladder D40% (adaptive) is less than bladder D40% (scheduled).

**(A)**	**Measure 1**	**Measure 2**	**W**	**z**	***p***
CTV1 V100% (adaptive)		CTV1 V100% (scheduled)	448.50	2.19	0.01
PTV1 D98% (adaptive)		PTV1 D98% (scheduled)	629.00	5.14	<0.001
PTV2 Boost D98% (adaptive)		PTV2 Boost D98% (scheduled)	564.50	4.56	<0.001
PTV2 Boost D0.1ccm <107% (adaptive)		PTV2 Boost D0.1ccm <107% (scheduled)	281.50	0.33	0.38
**(B)**	**Measure 1**	**Measure 2**	**W**	**z**	***p***
Bladder D40% (adaptive)		Bladder D40% (scheduled)	399.50	2.13	0.98
Bladder D100% (adaptive)		Bladder D100% (scheduled)	153.50	2.35	0.99
Bladder V40Gy (adaptive)		Bladder V40Gy (scheduled)	12.00	−2.12	0.02
Bladder V37Gy (adaptive)		Bladder V37Gy (scheduled)	321.00	0.40	0.66
Bowel Bag V30Gy (adaptive)		Bowel Bag V30Gy (scheduled)	3.00	1.34	0.96
Bowel Bag D5ccm (adaptive)		Bowel Bag D5ccm (scheduled)	146.00	1.53	0.94
Rectum D50% (adaptive)		Rectum D50% (scheduled)	424.00	1.79	0.96
Rectum D20% (adaptive)		Rectum D20% (scheduled)	533.50	3.58	1.00
Rectum V36Gy (adaptive)		Rectum V36Gy (scheduled)	253.50	−1.01	0.16
Penile bulb D50% (adaptive)		Penile bulb D50% (scheduled)	229.50	0.26	0.61

**Table 3:** Summary of couch shifts (in cm) applied to correct for target motion detected in CBCT2 following the adaptation process. The Ethos^®^ treatment couch supports corrections in three translational directions only. For patients in the supine position, a positive longitudinal shift indicates a target displacement in the superior direction, a positive lateral shift corresponds to a displacement to the left, and a positive vertical (height) shift reflects a displacement toward the anterior.

	Longitudinal (z) in cm	Lateral (x) in cm	Vertical (y) in cm
Mean	−0.01	−0.04	−0.01
Std. Dev.	0.16	0.10	0.21
Minimum	−0.68	−0.26	−0.72
Maximum	0.11	0.18	0.28
2.5th percentile	−0.48	−0.21	−0.58
25th percentile	0.00	−0.12	0.00
Median	0.03	−0.01	0.07
75th percentile	0.06	0.01	0.09
97.5th percentile	0.11	0.16	0.19

**Table 4:** Summarized treatment delivery times per period in minutes.

	Contouring/Adaptation [min]	QA Time [min]	Beam-on Time [min]	Interval Between CBCT1–CBCT2 [min]	Total Treatment Time [min]
Mean	12:11	05:27	05:22	17:38	30:17
Std. Dev.	05:14	01:23	00:44	05:02	05:49
Minimum	04:09	03:00	03:58	11:00	22:37
Maximum	24:51	08:42	06:39	28:35	43:07
25th percentile	07:59	04:35	04:43	16:00	25:07
Median	10:37	05:00	05:29	13:30	30:31
75th percentile	16:20	06:04	05:56	21:49	33:00

**Table 5:** Toxicity at eight weeks after completion of treatment according to CTCAE version 5.0. No grade ≥3 events were observed.

CTCAE Event	Diarrhea	Proctitis	Rectal Bleeding	Cystitis	Urinary Retention	Hematuria	Erectile Dysfunction	Fatigue
Grade 0	6 (87.5%)	3 (42.9%)	6 (85.7%)	3 (42.9%)	6 (85.7%)	7 (100.0%)	5 (71.4%)	7 (100.0%)
Grade 1	1 (14.3%)	1 (14.3%)	-	3 (42.9%)	1 (14.3%)	-	1 (14.3%)	-
Grade 2	-	3 (42.9%)	1 (14.3%)	1 (14.3%)	-	-	1 (14.3%)	-

**Figure A1:**
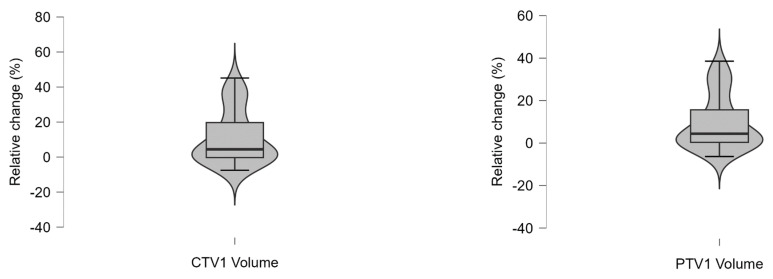

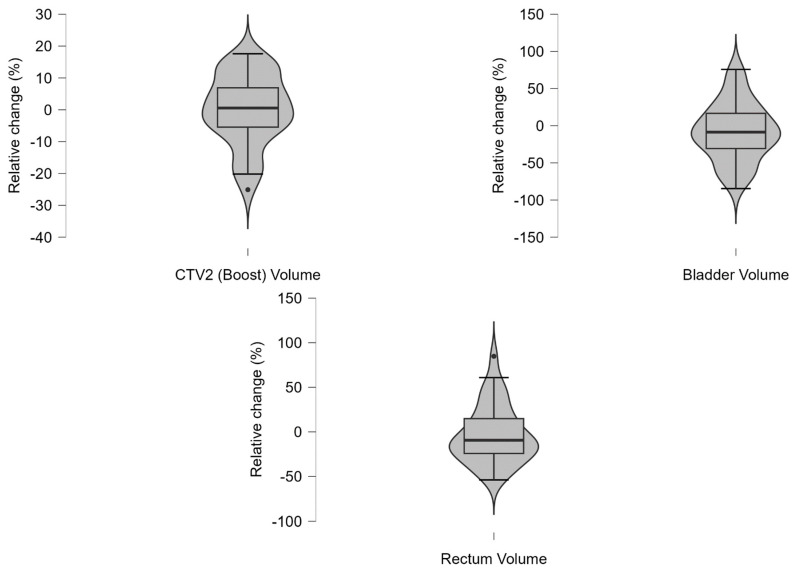
The corresponding value distribution of target and organs at risk (OAR) is shown in boxplots with violin elements.

## Data Availability

Research data are stored in an institutional repository and will be shared upon request to the corresponding author.

## Abbreviations

The following abbreviations are used in this manuscript:

AI	Artificial intelligence
CBCT	Cone beam computed tomography
CT	Computed tomography
CTCAE	Common Terminology for Adverse Events
CTV	Clinical target volume
DVH	Dose–volume histogram
GTV	Gross target volume
IMRT	Intensity-modulated radiotherapy
LINAC	Linear accelerator
OAR	Organ at risk
oART	Online-adaptive radiotherapy
PRO	Patient-reported outcome
PTV	Planning target volume
SIB	Simultaneous integrated boost
uhRT	Ultra-hypofractionated radiotherapy
VMAT	Volumetric modulated arc therapy

## Appendix A

### Appendix A.1

Planning template for ETHOS^®^ treatment planning system.

**Table A1:** Applied dose prescription for clinical target volumes (CTV).

Target	Dose Per Fraction	Total Dose
CTV1 Prostata	7.25	36.25
CTV2 Boost (SIB)	8.00	40.00

**Table A2:** Summarized planning features within the so-called ‘RT-intent’ template of the Ethos treatment planning software v2.0 (Varian, Palo Alto, CA, USA). Each structure serves as an influencer, with a hierarchical order determined by a given priority (P), where 1 is the most important. Priorities greater than 2 do not influence the planning algorithm (these are ‘report-only structures’).

Target/Organ at Risk	Dose/Volume Reference	Optimal	Mandatory	Priority
PTV1 7.25 Gy	D98%	>97%	≥95%	1
	D0.1cc	<42.8 Gy	≤44 Gy	1
	V95%	≥99%	≥95%	2
	Dmax	≤42.8 Gy	≤44 Gy	2
CTV1 7.25 Gy	V100%	≥99%	≥95%	1
	D98%	>98%	≥96%	1
CTV2 (Boost) = PTV2 8.00 Gy	D0.1%	<105%	≤107%	1
	D98%	>98%	≥95%	1
	D100%	>97%	≥95%	1
	V95%	>99%	≥95%	2
	D1%	<105%	≤107%	2
Rectum	V36 Gy	<0.8 cc	≤1 cc	1
	D50%	<15 Gy	≤18 Gy	2
	D20%	<25 Gy	≤29 Gy	2
Bladder	V40 Gy	<1 cc	≤1.5 cc	1
	V37 Gy	<5 cc	≤10 cc	1
	D40%	<15 Gy	≤18 Gy	2
	D100%	<2.6 Gy	≤3.6 Gy	2
Bowel Bag	V30 Gy	<0.1 cc	≤1 cc	1
	D5%	<16 Gy	≤18.10 Gy	2
External	Dmax	<42.8 Gy		2
Femoral head (L/R)	D5%	<13.5 Gy	≤14.5 Gy	2
	V30 Gy	< 9 cc	≤10 cc	3
Penile bulb	D50%	<27 Gy	≤29.5 Gy	2

### Appendix A.2

Target and organ at risk (OAR) volumes at planning CT

**Table A3:** Summary statistics of target and organ at risk (OAR) volumes at planning CT. CTV2 corresponds to the prostate volume.

Target and OAR Volumes					
	CTV1, i.e., Prostate + SV [cm^3^]	PTV1 [cm^3^]	CTV2 Boost, i.e., Prostate [cm^3^]	Bladder [cm^3^]	Rectum [cm^3^]
Mean	79.86	144.43	60.71	300.43	69.14
Std. Deviation	17.58	25.77	13.70	103.44	23.72
Minimum	60.00	114.00	51.00	148.00	32.00
Maximum	113.00	192.00	91.00	453.00	101.00
Q1	68.50	129.00	54.50	230.00	57.00
Median	78.00	142.00	55.00	346.00	63.00
Q3	85.50	152.50	59.50	348.00	87.00

### Appendix A.3

Dose–volume histogram (DVH) metrics.

**Table A4:** Dose–volume histogram (DVH) metrics of the reference plan for targets and organs at risk based on the planning CT (V, in cm^3^).

Descriptive Statistics										
	PTV1 D98%	PTV2 Boost D98%	PTV2 Boost D0.1ccm <107%	Bladder V37Gy	Bladder V40Gy	Bladder D100%	Bladder D40%	Rectum V36Gy	Rectum D20%	Rectum D50%
Mean	93.74	96.51	102.14	2.48	0.01	0.79	9.82	0.32	24.02	14.86
Std. Deviation	1.80	1.29	0.60	1.40	0.03	0.23	4.20	0.20	2.40	1.53
Minimum	90.30	94.88	101.30	1.11	0.00	0.47	3.01	0.12	19.85	13.58
Maximum	95.80	98.90	102.80	5.16	0.07	1.14	15.41	0.62	26.88	17.65
Q1	93.25	95.95	101.70	1.72	0.00	0.64	7.44	0.15	23.32	13.71
Median	93.90	96.20	102.30	1.84	0.00	0.79	10.55	0.32	23.94	14.03
Q3	94.85	96.85	102.60	2.89	5.00 × 10^−3^	0.94	12.46	0.45	25.41	15.67
	Bowel Bag V30Gy		Bowel Bag D5ccm	Penile bulb D50%		Femoral Head L D5%	Femoral Head L V30Gy	Femoral Head R D5%		Femoral Head R V30Gy
Mean	0.13		2.87	9.70		13.16	0.00	11.13		0.00
Std. Deviation	0.34		1.61	7.73		0.90	0.00	3.49		0.00
Minimum	0.00		1.87	2.11		12.07	0.00	4.03		0.00
Maximum	0.89		6.11	20.74		14.35	0.00	13.08		0.00
Q1	0.00		2.15	3.67		12.61	0.00	12.08		0.00
Median	0.00		2.25	7.54		12.93	0.00	12.46		0.00
Q3	0.00		2.51	15.29		13.87	0.00	12.69		0.00

### Appendix A.4

Summarized target and organ at risk (OAR) volumes.

**Table A5:** Summarized target and organ at risk (OAR) volumes (V, in cm^3^) including n = 35 fractions.

Target and Organ at Risk (OAR) Volumes (V)					
	V CTV1 [cm^3^]	V PTV 1 [cm^3^]	V CTV 2 Boost [cm^3^]	V Bladder [cm^3^]	V Rectum [cm^3^]
Mean	88.14	157.57	60.84	259.42	64.44
Std. Deviation	22.79	32.96	15.19	115.76	27.11
Minimum	63.70	119.00	38.20	69.90	29.00
Maximum	164.00	266.00	107.00	589.00	149.00
Q1	74.90	139.00	53.75	146.00	46.65
Median	81.80	149.00	57.50	267.00	56.30
Q3	92.40	163.50	61.45	331.00	72.65

### Appendix A.5

Relative inter-fraction volume changes.

**Table A6:** Descriptive characteristics of relative inter-fraction volume changes and corresponding violin plots: Comparison of relative volume changes (%) between planning-CT and first cone beam CT (CBCT1) at each fraction in all patients (n = 35 fractions). The violin plots, with superimposed boxplots, illustrate the distribution density of volume changes, including quartiles and the full data range.

Relative Volume Change (%)					
	Bladder Volume	Rectum Volume	CTV1 Volume	PTV1 Volume	CTV2 (Boost) Volume
Mean	−8.20	−3.37	10.56	9.23	0.05
Std. Deviation	39.24	32.54	14.98	12.58	11.05
Minimum	−84.57	−53.84	−7.56	−6.34	−25.10
Maximum	75.73	84.75	45.13	38.54	17.58
25th percentile	−30.76	−24.22	−0.30	0.35	−5.45
Median	−8.67	−9.38	4.42	4.40	0.55
75th percentile	16.64	14.85	19.70	15.65	6.88

### Appendix A.6

Dose–volume histogram (DVH) statistics.

**Table A7:** Dose–volume histogram (DVH) statistics of target volume and organ at risk (OAR) doses of adaptive and scheduled plans (per fraction).

Descriptive Statistics																
	Bladder D40%		Bladder D100%		Bladder V40Gy		Bladder V37Gy		Bowel Bag V30Gy				Bowel Bag D5ccm			
	Adaptive Plan	Scheduled Plan	Adaptive Plan	Scheduled Plan	Adaptive Plan	Scheduled Plan	Adaptive Plan	Scheduled Plan	Adaptive Plan		Scheduled Plan		Adaptive Plan		Scheduled Plan	
Std. Deviation	1.12	1.12	0.09	0.10	0.10	0.08	1.27	1.11	0.07		0.01		0.40		0.39	
Minimum	0.37	0.33	0.07	0.07	0.00	0.00	0.09	0.06	0.00		0.00		0.15		0.15	
Maximum	4.52	4.56	0.51	0.59	0.62	0.36	5.50	3.93	0.39		0.06		2.05		1.90	
Q1	1.15	1.16	0.13	0.12	0.00	0.00	1.23	0.92	0.00		0.00		0.28		0.29	
Median	2.20	1.90	0.18	0.16	0.00	0.00	1.46	2.05	0.00		0.00		0.44		0.43	
Q3	2.75	2.48	0.21	0.21	0.00	0.03	2.58	2.58	0.00		0.00		0.58		0.55	
	Rectum D50%		Rectum D20%		Rectum V36Gy		Penile bulb D50%		Femoral head L D5%		Femoral Head L V30Gy		Femoral head R D5%		Femoral Head R V30Gy	
	Adaptive Plan	Scheduled Plan	Adaptive Plan	Scheduled Plan	Adaptive Plan	Scheduled Plan	Adaptive Plan	Scheduled Plan	Adaptive Plan	Scheduled Plan	Adaptive Plan	Scheduled Plan	Adaptive Plan	Scheduled Plan	Adaptive Plan	Scheduled Plan
Mean	2.94	2.79	5.03	4.71	0.46	0.64	2.22	2.24	2.72	2.71	0.00	0.00	2.38	2.30	0.00	0.00
Std. Deviation	0.59	0.71	0.46	0.46	0.65	0.74	1.65	1.96	0.20	0.35	0.00	0.00	0.73	0.63	0.00	0.00
Minimum	1.20	0.86	4.17	3.64	0.04	0.00	0.43	0.40	2.28	2.32	0.00	0.00	0.81	0.93	0.00	0.00
Maximum	4.45	3.83	6.31	5.45	2.84	3.08	5.67	6.17	3.30	3.58	0.00	0.00	3.37	2.80	0.00	0.00
Q1	2.74	2.33	4.79	4.49	0.15	0.08	0.62	0.61	2.62	2.44	0.00	0.00	2.46	2.34	0.00	0.00
Median	2.78	3.04	5.00	4.74	0.20	0.35	2.25	1.58	2.71	2.63	0.00	0.00	2.63	2.53	0.00	0.00
Q3	3.30	3.31	5.17	5.08	0.38	0.83	3.24	3.46	2.79	2.86	0.00	0.00	2.75	2.69	0.00	0.00

### Appendix A.7

Correlation analysis of intra-fractional motion.

**Table A8:** Spearman’s correlation analysis of absolute couch shifts with different periods of the treatment process. No statistically significant correlation (*p* < 0.05) was observed.

Spearman’s Correlations				
Variable		longitudinal couch shift (z, absolute values [cm])	lateral couch shift (x, absolute values [cm])	vertical couch shift (y, absolute values [cm])
Interval between CBCT1–CBCT2 [s]	Spearman’s ρ	−0.022	0.011	−0.082
	*p*-value	0.902	0.950	0.642
	Lower 95% CI	−0.352	−0.323	−0.404
	Upper 95% CI	0.314	0.343	0.259
Contouring/Adaptation time [s]	Spearman’s ρ	−0.025	0.002	−0.181
	*p*-value	0.886	0.991	0.297
	Lower 95% CI	−0.355	−0.332	−0.485
	Upper 95% CI	0.311	0.335	0.162
QA time [s]	Spearman’s ρ	−0.075	−0.077	0.129
	*p*-value	0.670	0.662	0.460
	Lower 95% CI	−0.398	−0.400	−0.213
	Upper 95% CI	0.265	0.263	0.443
